# Supplementary material for: Insights into SusCD-mediated glycan import by a prominent gut symbiont
Source: Nat Commun. 2021 Jan 4;12:44. doi: 10.1038/s41467-020-20285-y (PMC7782687; doi:10.1038/s41467-020-20285-y)
Supplement: Supplementary file 1 — Supplementary Information [file 41467_2020_20285_MOESM1_ESM.pdf]

Supplementary Information

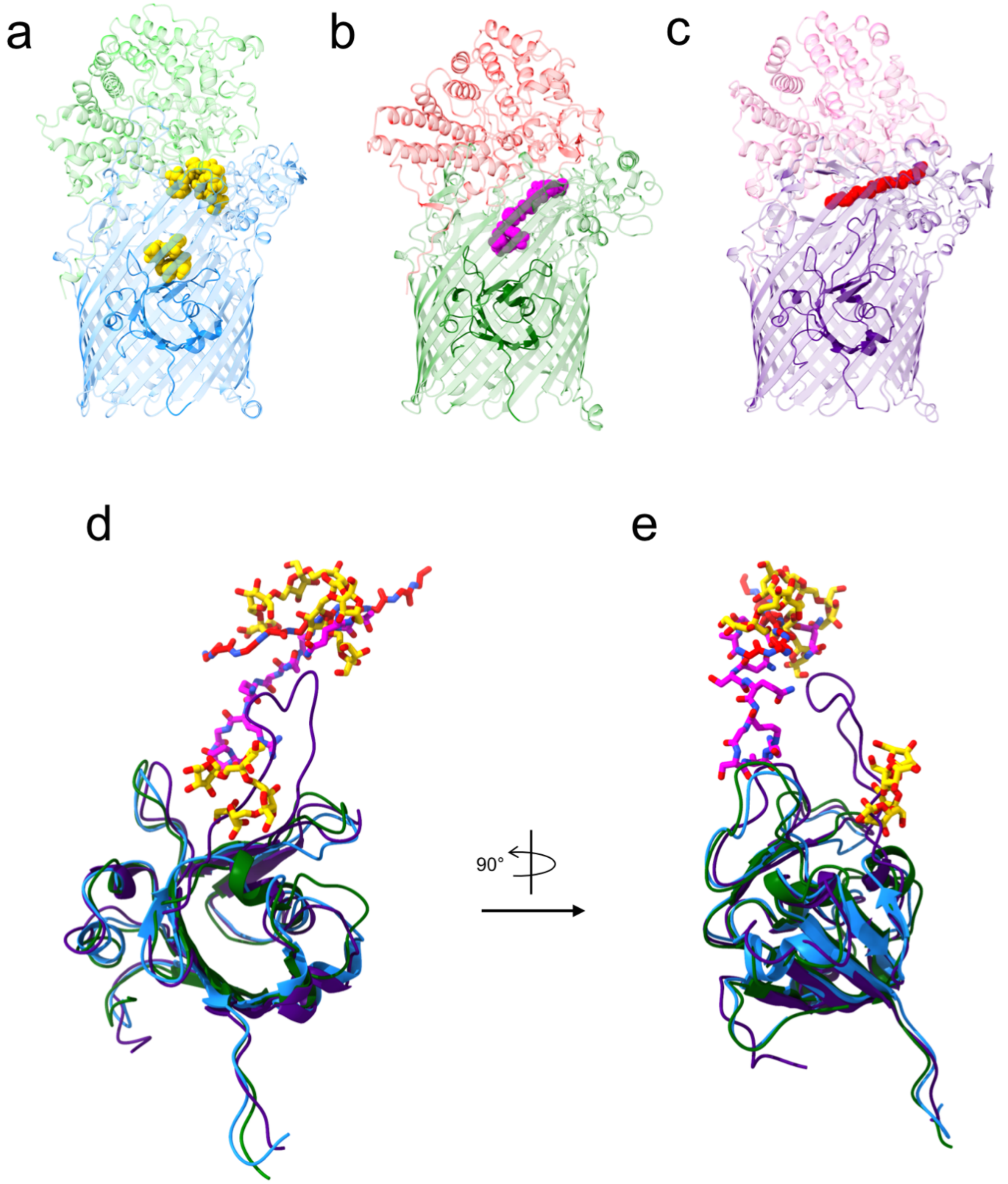

Supplementary Figure 1 Comparison of SusCD ligand binding sites. **a-c**, Cartoon representations of (**a**) Bt1762-63 with bound FOS (yellow), (**b**) *Porphyromonas gingivalis* RagAB with bound peptide (magenta; PDB ID 6SLN), and (**c**) Bt2263-64 with bound peptide (red; PDB ID 5FQ8). The bottom panels show close-up views of the plugs and ligands from the membrane plane, rotated 90° between **d** and **e**.

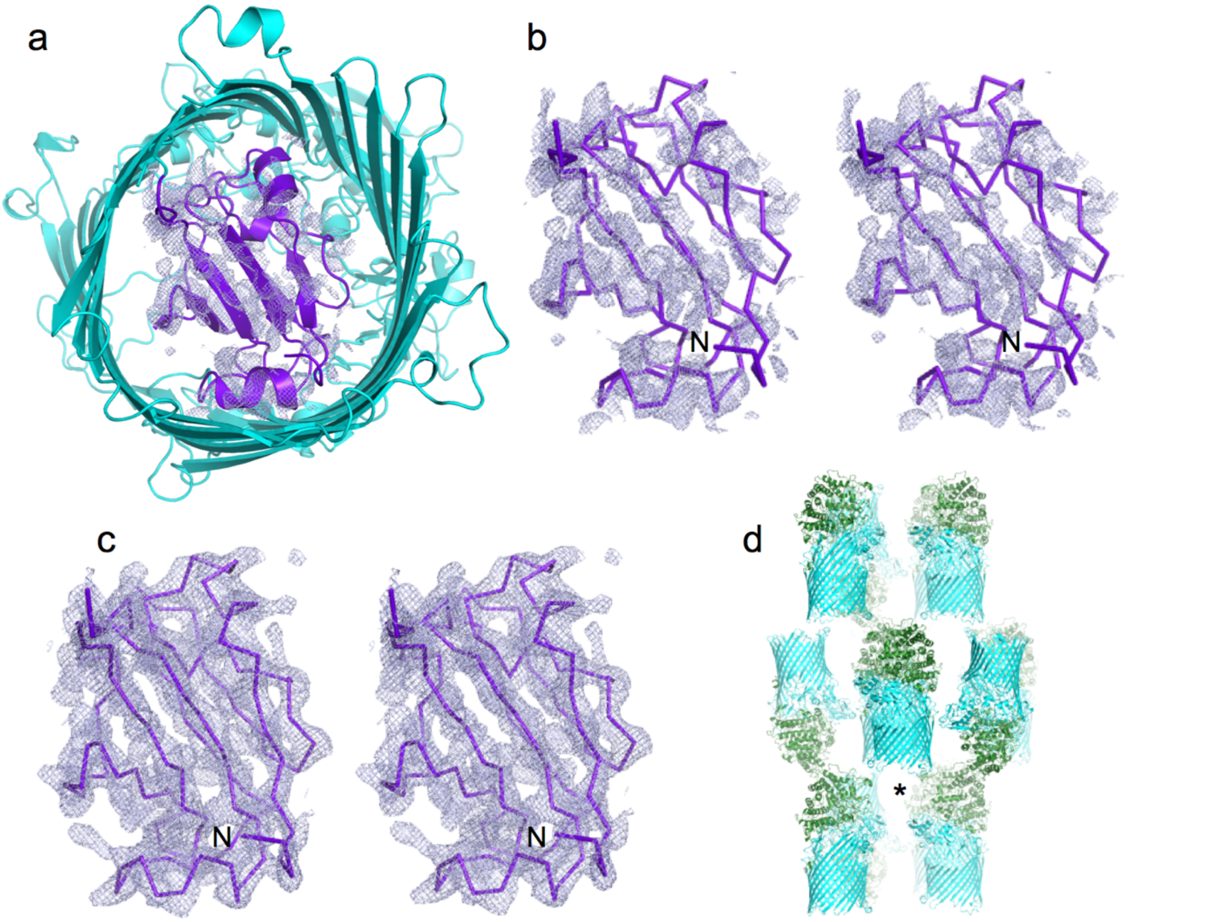

Supplementary Figure 2 Crystal structure of apo Bt1762-63 showing poor density for the plug. **a**, Cartoon viewed from the periplasmic space, with the plug domain of the substrate-bound state superposed and coloured purple, showing poor 2Fo-Fc density for the plug (σ = 0.8, carve = 2). **b**, Stereo view of plug density of apo Bt1762-63 with the Cα ribbon superimposed in the same orientation as (**a**). The N-terminus is labelled (N). **c**, Stereo Cα ribbon with plug density for substrate-bound Bt1762-63 (σ = 1.5, carve = 2). **d**, Crystal packing of apo Bt1762-63. The large space within the crystal that could contain an ejected plug domain is indicated with an asterisk.

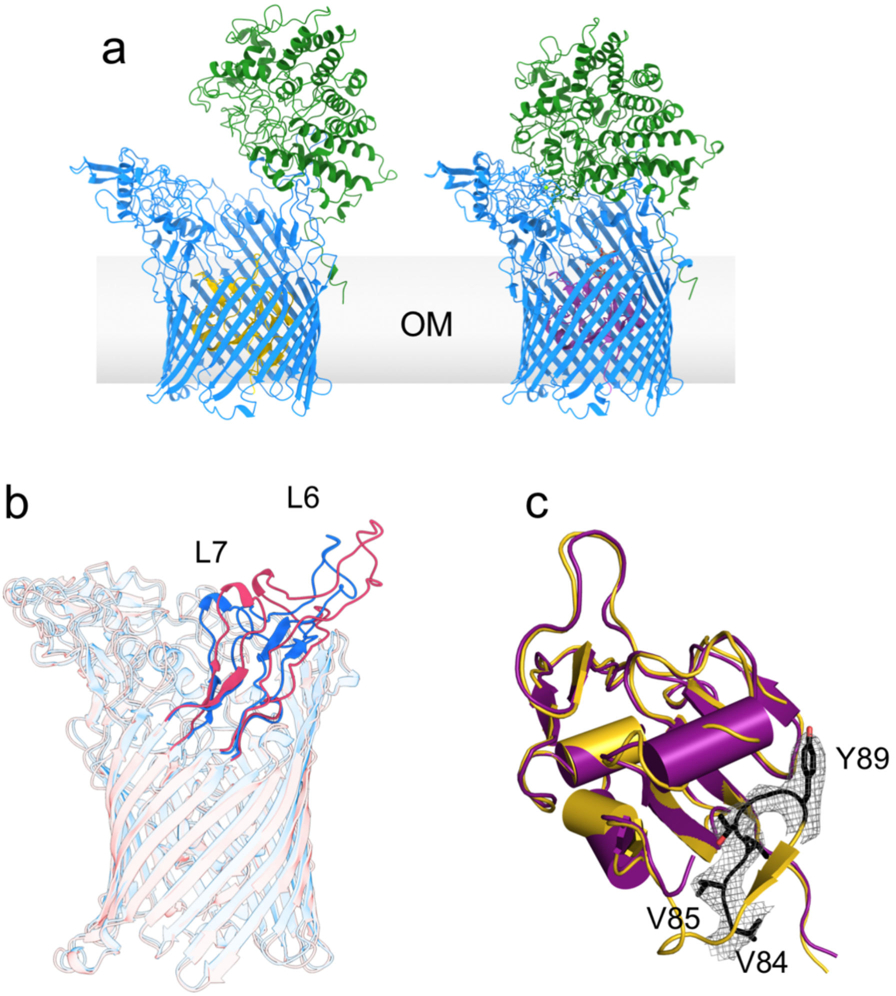

Supplementary Figure 3 Differences between the open and closed structures of Bt1762-63. **a**, Cartoon models of the individual open (left) and substrate-bound, closed (right) transporter structures derived by cryo-EM and X-ray crystallography, respectively. For clarity, transporters are shown as monomers. **b,** Superposition of open (red) and closed states (blue) of Bt1763, with both hinge loops L6 (Hinge 2) and L7 (Hinge 1) highlighted. **c**, Overlay of plug domains from open (gold) and closed (purple), substrate-bound forms of the transporter. The Ton box (^{82}DEVVVTG^{88}) is visible only in the open state and is colored black, with sidechains displayed as sticks. The equivalent region in the closed structure is not visible and is therefore assumed to be disordered and likely protrudes from the barrel, leaving the Ton box accessible to TonB. The visible density for the N-terminus starts at residue 96 of the "closed" plug and at residue 84 for the "open" plug.

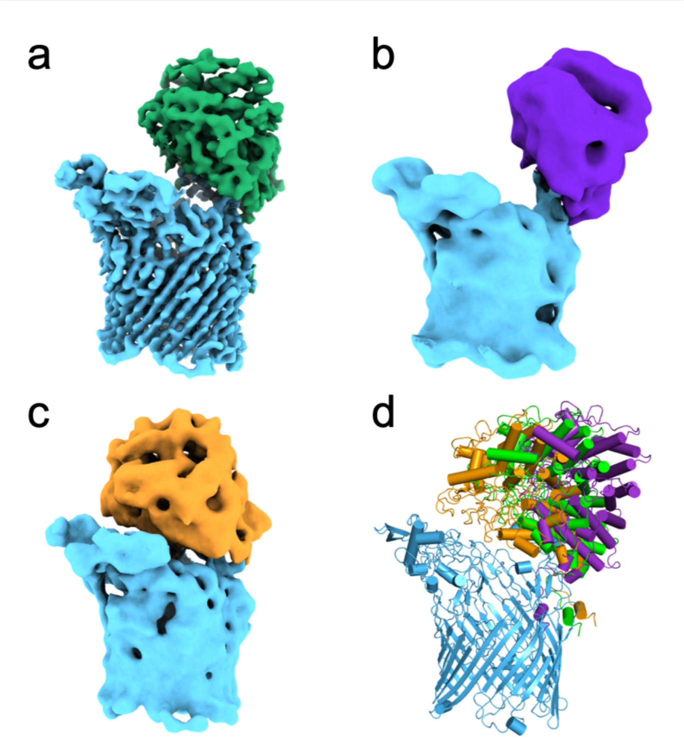

Supplementary Figure 4 Variability in the position of Bt1762 observed in the open states of the transporter visualised by cryo-EM. **a**, Predominant open position observed in both the principle open-open and open-closed states. Less populated classes with wide open (**b**) and barely open (**c**) lid positions were also observed. **d**, Overlaid rigid-body fits of Bt1762 into the maps shown in **a-c**. EM density is filtered by local resolution and monomers are shown for clarity. The number of particles assigned to wide-open and barely-open states are 9,903 and 12,637 respectively.

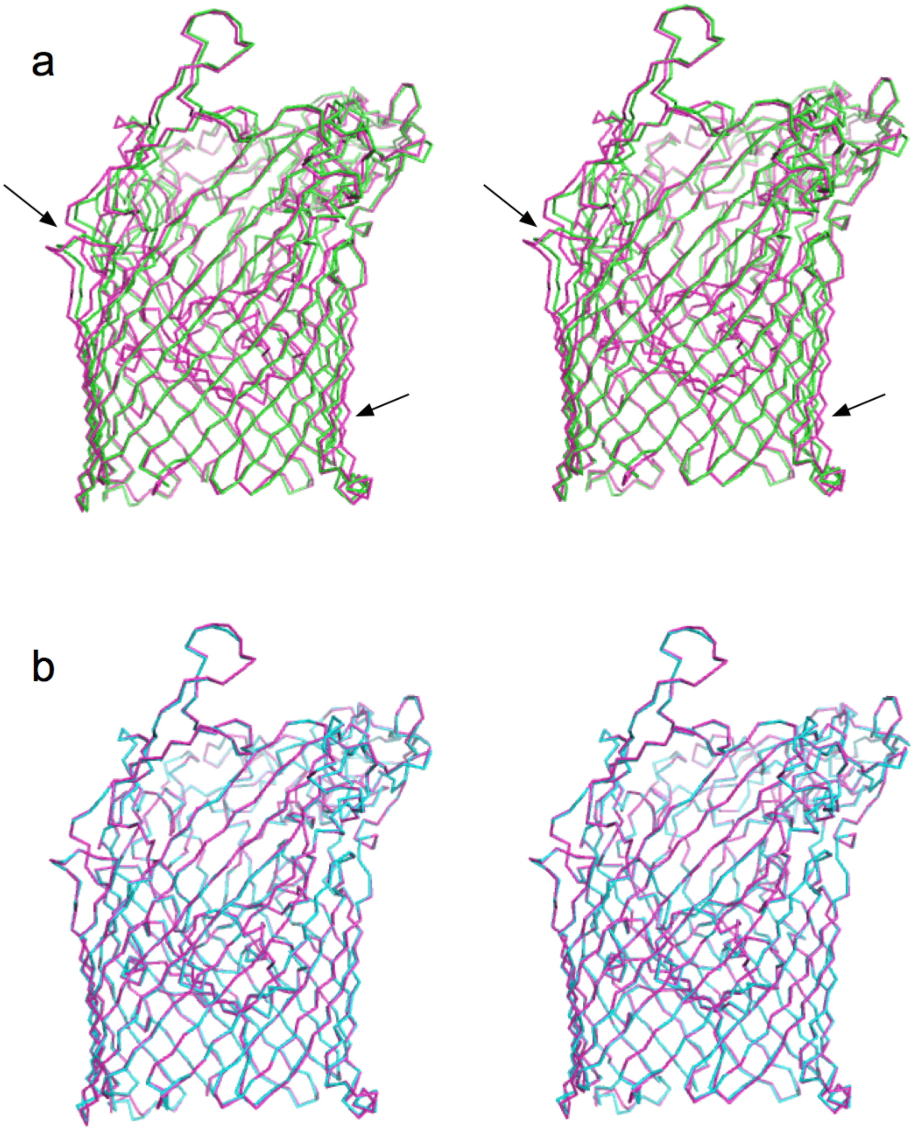

Supplementary Figure 5 Differences in barrel shape for apo- and substrate-bound Bt1763. **a,b** Stereo ribbon superpositions for apo-Bt1763 (green; PDB 6Z8I) and substrate-bound Bt1763 (magenta; PDB 6ZAZ) (**a**) and for both substrate-bound Bt1763 structures (PDB ID 6Z9A and 6ZAZ) (**b**). Superpositions were generated in COOT via secondary structure matching for Bt1763. The arrows highlight some of the differences within the barrels. Both structures of substrate-bound Bt1763 are identical (**b**).

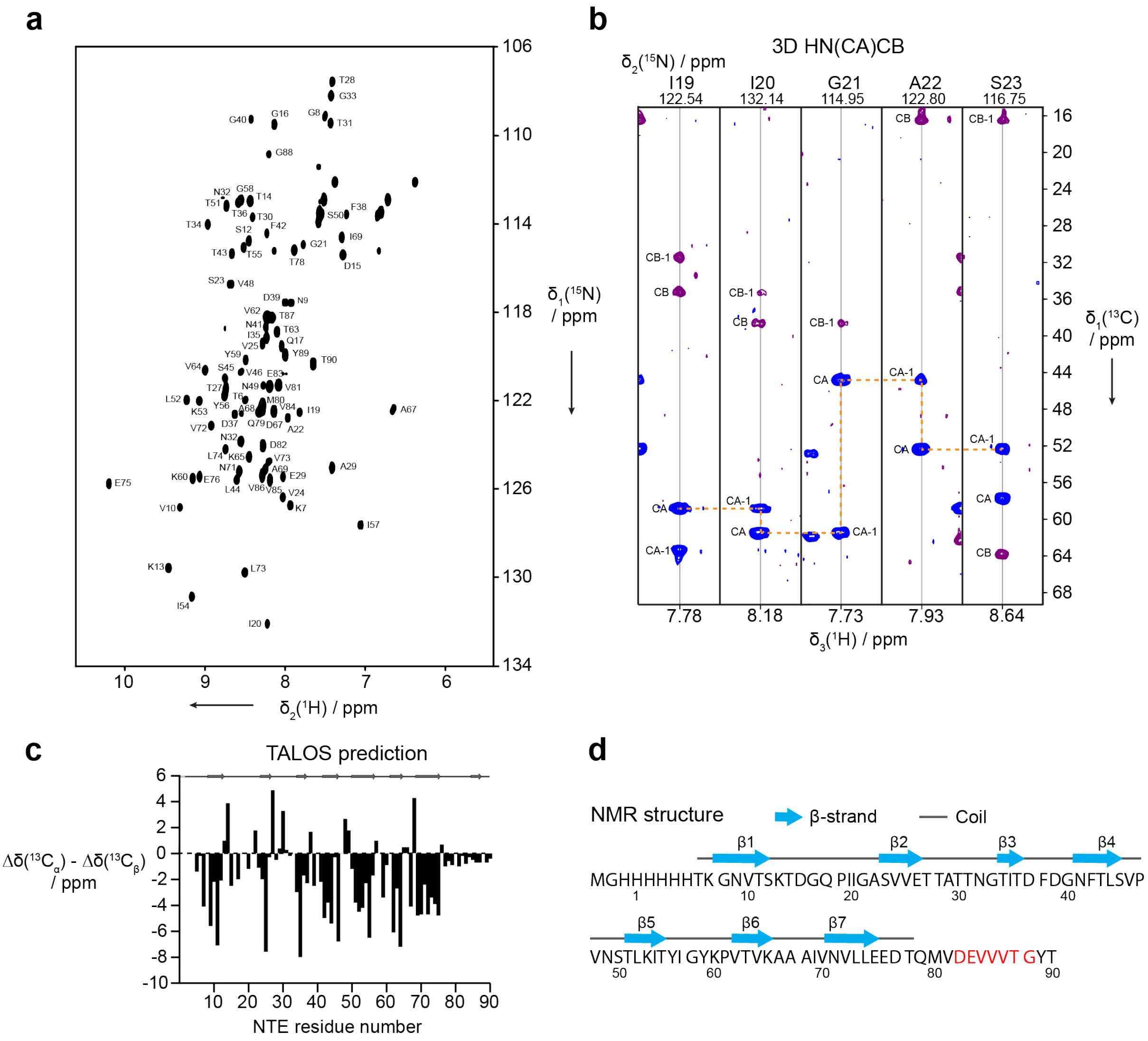

Supplementary Figure 6 NMR backbone assignment and secondary structure of the Bt1763 NTE domain. **a**, 2D [^{15}N,^{1}H]-HSQC spectrum of NTE at 20 °C and pH 7.5. Sequence-specific resonance assignments are indicated. **b**, Example of backbone strips taken from the 3D HN(CA)CB experiments for residues I19–S23. Positive and negative intensity is shown in blue and purple, respectively. The C_α backbone walk is indicated by an orange dashed line. **c**, Secondary chemical shifts relative to random coil values. Consecutive stretches of negative values indicate β-sheet secondary structure. The result of a TALOS secondary structure prediction using the chemical shifts from backbone resonance assignment is plotted on top. **d**, Sequence of the recombinant form of the NTE domain including the N-terminal His_6-tag. β-sheet secondary structure distributions over the NTE amino acid sequence as determined by solution NMR spectroscopy are indicated. The Ton box is shown in red.

```
# Job:
# Query: s001A
# No:  Chain    Z    rmsd lali nres  %id PDB  Description
   1:  5aq0-B 10.2  1.5   70    82   23   MOLECULE: CARBOXYPEPTIDASE D;
   2:  1uwy-A  7.9  1.7   67   393   22   MOLECULE: CARBOXYPEPTIDASE M;
   3:  5hbb-C  7.9  3.9   76   267   13   MOLECULE: CELL SURFACE PROTEIN SPAA;
   4:  4uzg-A  7.8  2.5   72   277   18   MOLECULE: SURFACE PROTEIN SPB1;
   5:  5k39-B  7.8  5.7   76   153   24   MOLECULE: CELLULOSOME ANCHORING PROTEIN COHESIN REGION;
   6:  3e8v-A  7.7  2.1   70    82   21   MOLECULE: POSSIBLE TRANSGLUTAMINASE-FAMILY PROTEIN;
   7:  6fwv-A  7.6  7.8   82   522   17   MOLECULE: COLLAGEN ADHESION PROTEIN;
   8:  4oq1-A  7.3  2.7   71   331   14   MOLECULE: CELL WALL SURFACE ANCHOR FAMILY PROTEIN;
   9:  3kpt-B  7.1  3.4   74   355   15   MOLECULE: COLLAGEN ADHESION PROTEIN;
  10:  4p0d-A  6.7  2.6   72   470   14   MOLECULE: TRYPSIN-RESISTANT SURFACE T6 PROTEIN;
  11:  1r6v-A  6.6  9.4   81   671   12   MOLECULE: SUBTILISIN-LIKE SERINE PROTEASE;
  12:  5n2j-B  6.5  2.3   73  1381   12   MOLECULE: UDP-GLUCOSE-GLYCOPROTEIN GLUCOSYLTRANSFERASE-LIKE
  13:  4fl4-I  6.3  2.3   73   309   19   MOLECULE: GLYCOSIDE HYDROLASE FAMILY 9;
  14:  4jdz-B  6.2  5.2   77   445   14   MOLECULE: SER-ASP RICH FIBRINOGEN/BONE SIALOPROTEIN-BINDING
  15:  1eo2-B  6.2  5.2   73   238   15   MOLECULE: PROTOCATECHUATE 3,4-DIOXYGENASE ALPHA CHAIN;
  16:  6fb3-A  6.2  6.9   75  1836   24   MOLECULE: TENEURIN-2;
  17:  4eiu-A  6.2  3.3   77   242   17   MOLECULE: UNCHARACTERIZED HYPOTHETICAL PROTEIN;
  18:  2y1v-A  6.1  3.5   73   604   18   MOLECULE: CELL WALL SURFACE ANCHOR FAMILY PROTEIN;
  19:  5z0z-B  6.1  2.8   73   450   16   MOLECULE: PILUS ASSEMBLY PROTEIN;
  20:  3uaf-A  6.0  4.0   70   117   20   MOLECULE: TTR-52;
  21:  3pf2-A  5.8  2.7   71   317   18   MOLECULE: CELL WALL SURFACE ANCHOR FAMILY PROTEIN;
  22:  2ww8-A  5.6  6.8   81   815   17   MOLECULE: CELL WALL SURFACE ANCHOR FAMILY PROTEIN;
  23:  5z5m-A  5.6  2.6   69   273   12   MOLECULE: PREDICTED PROTEIN;
  24:  5vxt-B  5.6  2.2   70   312   24   MOLECULE: CATECHOL 1,2-DIOXYGENASE;
  25:  4iyk-A  5.6  5.9   76   210    8   MOLECULE: UNCHARACTERIZED PROTEIN;
  26:  4z8w-A  5.5  3.0   70   125   23   MOLECULE: MAJOR POLLEN ALLERGEN PLA L 1;
  27:  6ts2-D  5.5  2.2   68  1123   13   MOLECULE: UDP-GLUCOSE-GLYCOPROTEIN GLUCOSYLTRANSFERASE-LIKE
  28:  6jch-A  5.2  2.5   70   327   17   MOLECULE: PILUS ASSEMBLY PROTEIN;
  29:  5xyr-A  5.2  2.4   62  1380    8   MOLECULE: CHEMOKINE PROTEASE C;
  30:  2x5p-A  5.2  2.4   67   104   16   MOLECULE: FIBRONECTIN BINDING PROTEIN;
```

Supplementary Figure 7 DALI analysis of the NTE. The thirty best hits are shown.

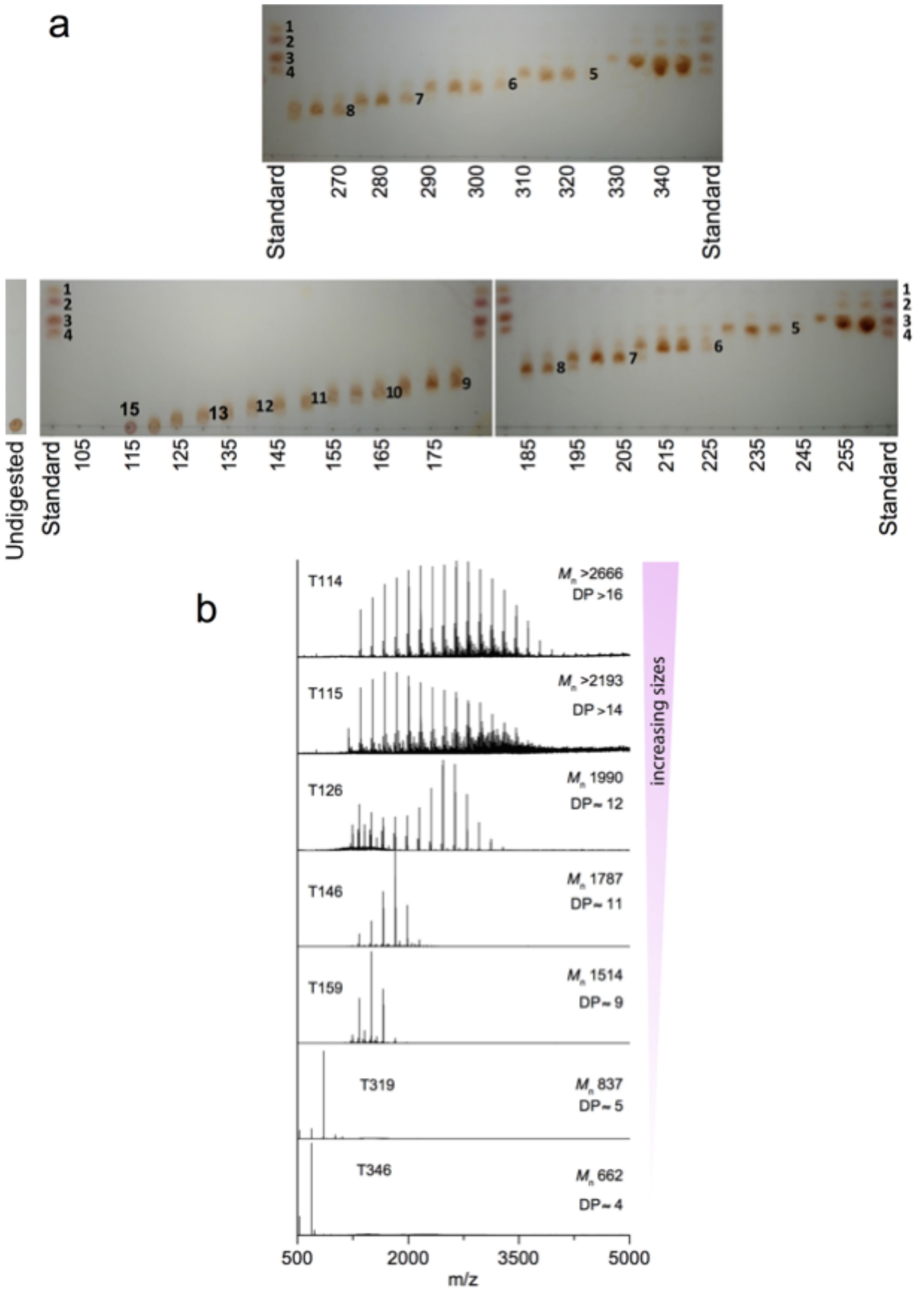

Supplementary Figure 8 Characterisation of β2,6 fructo-oligosaccharide (FOS) fractions. **a**, TLC analysis of digested *Erwinia* levan fractions from two different runs (top and bottom) after separation on a P2 SEC column. Approximate DP of FOS in each fraction are indicated. A sample of undigested levan is shown on the left. The standard consisted of β2,1 FOS1-4 (Fructose, sucrose, kestotriose, kestotetraose; Megazyme). **b**, Representative mass spectra of *Erwinia* FOS fractions from (**a**). The average molar mass is shown (M_n), based on the intensity-weighted signal intensities in the spectra. Source data are provided as a Source Data file.

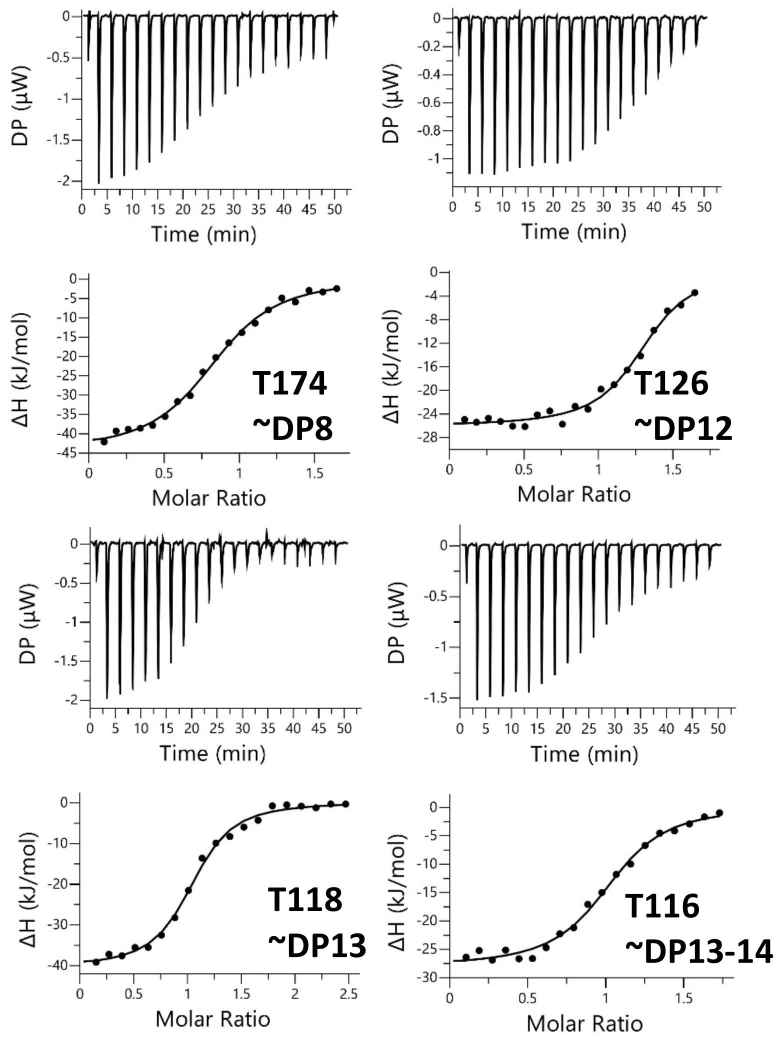

Supplementary Figure 9 ITC traces showing FOS binding to Bt1762-63 SusCD. β2,6 FOS fractions from SEC of partially digested *Erwinia* levan were titrated into purified Bt1762-63 SusCD complex (25 μM) in 100 mM Hepes, pH 7.5 containing 0.05% LDAO. The identity of the fraction used is indicated (T), as is the approximate DP of the main FOS species present in each fraction as determined by MS (Supplementary Fig. 8). The upper parts of each titration show the raw heats of injection and the lower parts the integrated heats, fit to a one set of sites model.

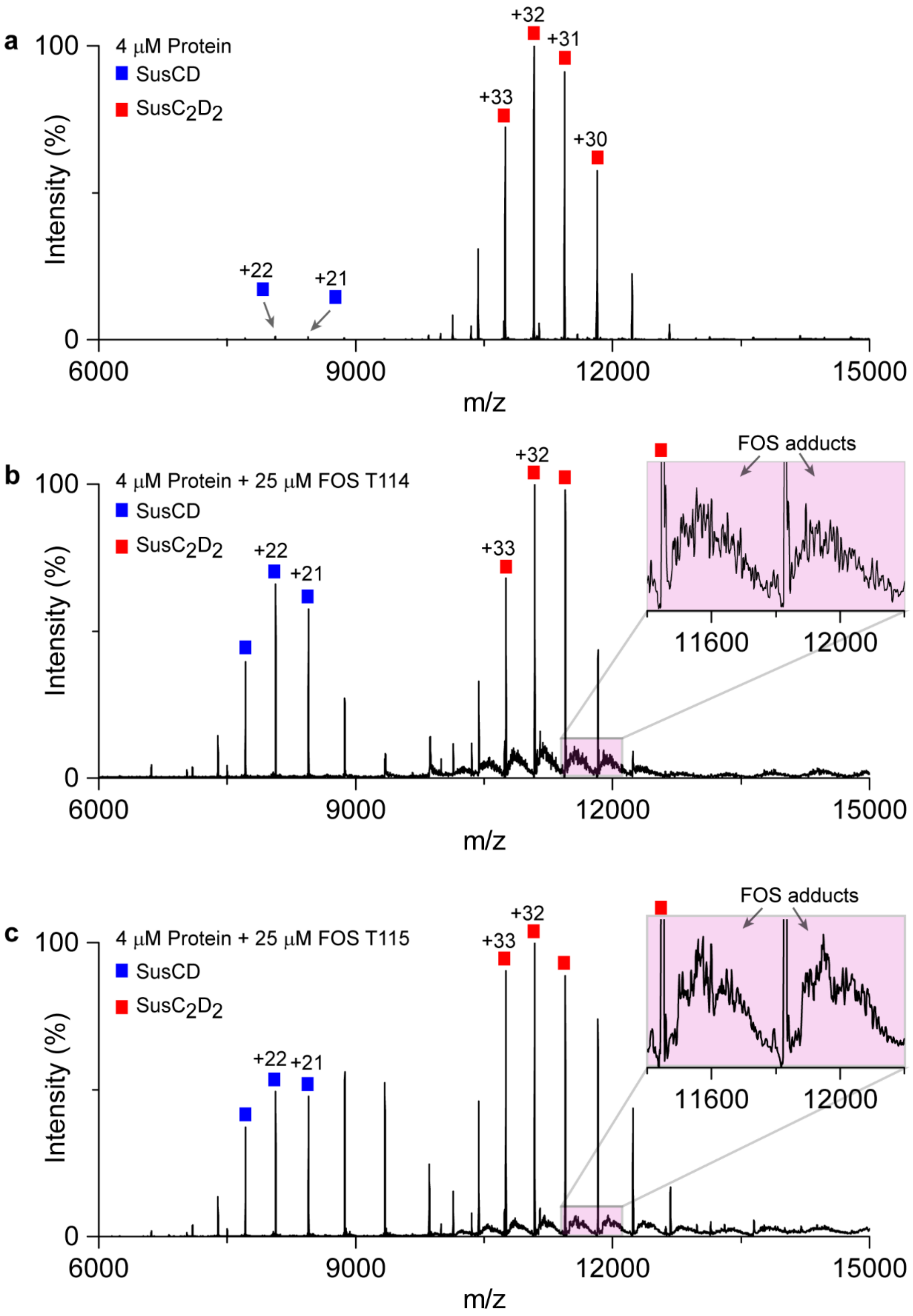

Supplementary Figure 10 Native mass spectra of Bt1762-63 without **(a)** and with long-chain FOS (**b**; T114, **c**; T115). Inserts, zoomed-in view of peaks corresponding to bound FOS molecules.

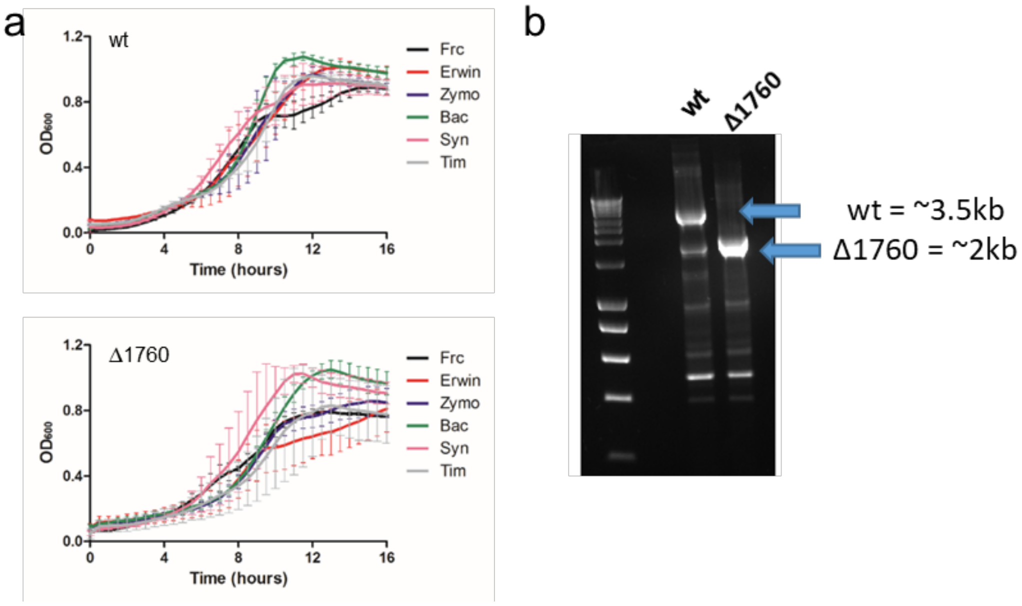

Supplementary Figure 11 Growth of *B. theta* wild-type and Δ1760 strains on different levans. **a**, *B. theta* wild type (wt) and Bt1760 deletion (Δ1760) strains were grown in minimal media with a range of levans as the sole carbon source. Levans used; Erwin = *Erwinia herbicola*, Zymo = *Zymomonas mobilis*, Bac = *Bacillus* sp., Syn = *in vitro* synthesised using levansucrase, Tim = Timothy grass. Frc = fructose. Growth curves show mean (solid line) +/- SD of triplicate wells and are representative of 3 independent experiments. **b**, PCR analysis of genomic DNA isolated from stationary phase cells of the *Erwinia* levan cultures. Primers flanking the *Bt1760* gene were used for amplification. This experiment was carried out once. Source data are provided as a Source Data file.

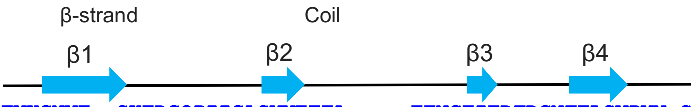


```
BT1763          LWGQNITVKGNVT--SKTDGQPIIGASVVETTA-----TTNGTITDFDGNFTLSVPVN-S     75
BT2264          ------------------------------------------------------------     0
PG0185 RagA     AMAQNRTVKGTVI--SSEDNEPLIGANVVVVGN-----TTIGAATDLDGNFTLSVPANAK     70
BT0268          -----------------------------------------MVIILPNFFLQKPVCRL     17
BT0272          VRAQDIPVSGVVK--DKSNNSTLPYASVIVKNESGKTVPQLSTTTDDNGRFSTKVKSG-Y     77
BT0362          WAQDAKVLKGRI---VNAEGEPIAGAVVNVAEA-----SR-IALSDKDGFFTLKNVKPAD     82
BT3090          AFAQQITVKGHV---VDATGEPVIGASVIEGK------STNGTITDIDGNFSLNVSAN-S     76
BT3332          FVLAQVLVKGTV---KDNLGEGVPGASVQVKG------TSQGTITDLDGKFTLNIPQKNA     64
BT3680          FAQGGIDVAGIV---LDEQGQELIGVSVQIKGK-----QGVGVVTDFDGRFKITGVPAGS     69
BT3702          AFAQQITVKGIV---KDTTGEPVIGANVVVKG------TTTGTITDFDGNFQLSAKQG-D     73
BT3983          STQQQQKVTGKV---VDANNEPLIGVSVLEKG------TTNGTITDFDGNYTLVVTGSNA     171
BT4114          VSAQTITLNGNV---KDTTGEPIIGASIVEKGN-----TTNGTITDLDGNFSLKVPAN-A     73
BT4121          ANAQTRKVTGQI---VDESGQPIIGATIRLQD------ATTGTITDIDGHFSLNVPDG-K     81
BT4164          AGQVQKVISGTVTELFGKTAEPLVGVNVNLVNNQNR--SLGGGITNLNGQYNVKVPEGEK     80
BT4168          WGQGGQKLTGKI---LSATNQPVEGAIVTVLDT-------MNVTTNKEGAFQFEVKDLSK     71
BT4660          LHAQNATVKGVI---VDETDTPLIGATVQVKG------TATGSITDFDGNYTIKANKG-A     76
BT4671          AQQGGKKMTGQV---IDENKEPMIGVSILIVG------TSTGTVTDFDGNYTLNVPKDSK     80
BACOVA_02096    IQQQSVKIKGRV---TDASGEPVIGANVVQKG------TTNGTITDLNGDFTLTVSQG-A     86
BACOVA_02652    APSQNLKVSGIVT--SATDGEPLIGVSVQVKG------TSTGTITDLNGKYTLNVSTG-Q     83
BACOVA_02742    IQQQSNKVTGKV---SD-ATGPIIGASVVEKG------TANGTITDLDGNFSLNVKAG-A     85
BACOVA_03426    SAQKGITVRGTV---LDSNGETIIGASVTLKGN-----NSVGTISDIDGNFVLTVPSEKS     79
BACOVA_03428    ------------------------------------------------------------     0
BACOVA_04393    --AQTVSVTGVV---KDASGEPIIGASVVEAG------TTNGIVTDLDGNFKLNVSAK-G     73
BACOVA_04505    LWGQNITVKGNVT--SKTDGQPIIGASVVQNDS-----KSTGTITDLDGNFTLSVPTN-A     75
```

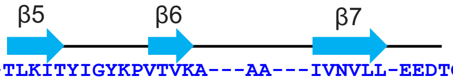


```
BT1763          --TLKITYIGYKPVTVKA---AA---IVNVLL-EEDTQMVDEVVVTGYTTQR-KA-----     120
BT2264          ------------MQTQEV-AIKP---NLKVVL-RSDAQQIDEVVVTAMGIKRSEK-----     38
PG0185 RagA     --MLRVSYSGMTTKEVAI---AN---VMKIVL-DPDSKVLEQVVVLGYGTGQKLS-----     116
BT0268          --LAVVGFLGFSGAIASQSSSPANIDSVKTYM---KKNSFEKNVGSRFVTNR-SI-----     66
BT0272          --RLVFSFLAFDSLSVKVTKPSE---RMQIYL-SPTENMLDETVVVGFKRVS-KA-----     125
BT0362          --ELYVSSVGYLPATAIADF-DE---NFKIVM-DADLDEYAHTTPLPFNRKP-KK-----     129
BT3090          --ALTISFVGYKTQTVSV-NGKT---ALKVTL-QEDTEVLDEVVVVGYGTMK-KS-----     123
BT3332          --TLVISFIGYVTVEQKA-DSQK---PMVITL-KEDTKTLDEVVVVGYQEVR-RR-----     111
BT3680          --TLVFSYIGYETREIKYTATKL---KEKIAL-KEAVNEFDEVVVVGRDTQR-KV-----     117
BT3702          --IIVVSFIGYQPQELPV-AA-----QMNVIL-KDDTEILDEVVVIGYGQVK-KN-----     118
BT3983          --VLQFSYVGYQTLERAV-AGKT---AINITL-KEDAQVLDEVVVTALGIKRSEK-----     219
BT4114          --TVVISYIGMKTQEIAI-KGKS---KIDVTL-SDDAKALDEVVVIGYGTAK-RK-----     120
BT4121          --KVVISYIGYLKQVILP-K-GD---TLKVIL-QEDNQKLDEVVVVGYGSMK-QK-----     127
BT4164          DLTIVYSYIGMKTKRIKY-TGQT---LLNVTL-ESESMAVDEVVVSARRLNRNDLGISDK     135
BT4168          AGEISVWAPGYFSVKQLIRE-RS---NIVITLIPENQYKYNETMILPFRREG-EMQLE--     124
BT4660          --VITFSYIGYKTQEIKF-TGQS---PLNVKM-IPDNQTLDEVVVVGYGTMK-RS-----     123
BT4671          --ELQFSYVGYETKVITIPVNSN---VLNVQM-KSDSQVLSDVVIIGYGTQR-KS-----     128
BACOVA_02096    --VLQVSFIGYKQQEVSLKNGQA---QVTVVL-KDDAELLDEVVVVGYGTMK-KR-----     134
BACOVA_02652    --TLVFSYIGFMEQQV-V-ATKP---VINVVL-KEDTKTLDEVVVVGYGTMK-RS-----     129
BACOVA_02742    --TLVVSYVGYKSEE-VK-AGRG---PLNITL-KEDAKALDEVVVTALGIKRERK-----     132
BACOVA_03426    --VLIVSYVGMKPQEVKVSSKGM----IKVTL-EDDTKQLEEVVVVGYGQQK-KA-----     126
BACOVA_03428    -MNRKFIYIGCTVFAMSL---------LSMTGVQAQEESKDSLVNVAFGKVA-QE-----     44
BACOVA_04393    --SLKISFIGYQTQTIPV-AGKK---QFDITL-KEDAKVLDEVVVVGYGQMK-RS-----     120
BACOVA_04505    --LLAISYIGYKEVIIAA---KP---SLKIVM-EEDAKMIDEVVVTGYMAEK-KA-----     120
```

```
BT1763          DLTGAVSVVKV-DEIQK-QGENNPVKALQGRVPGMNITADGNPSGS-ATV-R------IR     170
BT2264          ALGYAATSVGG-EKIAE-SRTSDVMSSLAGKIAGVQISSTSSDPGASNSV-I------IR     89
PG0185 RagA     TVSGSVAKVSS-EKLAE-KPVANIMDALQGQVAGMQVMTTSGDPTAVASV-E------IH     167
BT0268          DSFGVSDTVDI-KMLQR-SQFLSIQQLLKGNVPGVYVQENNGEPGTIQSM-L------VR     117
BT0272          AVTASVTVIKA-EDLVN-TPVANPMELLQGRVPGLNIQMNNGTPGGLPSF-S------IR     176
BT0362          FVTESTSIVTG-EELEK-HPVTVLQNAFTSTVTGVETYEAQSEPGWSETAMY------IR     181
BT3090          DLTGAVSSVGV-KDIKD-SPVANIGQAMQGKVSGVQII-DAGKPGDNVTI-K------IR     173
BT3332          DLTGSVAKANM-ADVLT-APVASFDQALGGRIAGVNVTSGEGMPGGNMSI-V------IR     162
BT3680          SVVGAITNVDP-AGIQA--PAVSVSNMLGGRVPGIIAVTRSGEPGNNFSEFW------IR     168
BT3702          DMTGSVMAIKP-DELSK-GITTNAQDMLSGKIAGVSVISNDGTPGGGAQI-R------IR     169
BT3983          ALSYNVQQVNA-DAVTT-NKDPNFINSLSGKVAGVNINASSSGVGGVSKV-V------MR     270
BT4114          DITGSVATVNA-EALTV-VPVASATEALTGKMAGVQITTTEGSPDAEMKI-R------VR     171
```

```
BT4121          NITGSVSTISA-EELED-LPVSNLSEALQGMVNGLNVQLGSSRPGTNANEVYIRQNRTFT    185
BT4164          EMVSATQKVDMEKLIAA-APVVSIEEALQGQLGGVDIVL-GGDPGSRSAI-R------IR    186
BT4168          DYTAAT-NIAK-KDFMP--GTTKIDRALTGQVAGLQVKRSSGMPGEGSYY-N------LR    173
BT4660          DLTGSVASIAA-KDVEG-FKTSSVAGALGGQIAGVQITSTDGTPGAGFSI-N------IR    174
BT4671          DLTGSVASVGT-KDFNK-GMVSSPEELVNGKIAGVQIVNGGGSPTSVSTI-R------IR    179
BACOVA_02096    DLSGAVSQIKS-DDLMK-GNPTDLSKGLAGKIAGVQVNQSDGAPGGGISI-Q------IR    185
BACOVA_02652    DLTGSVVSVTG-DELKK-SVVTSLDQALQGRAAGVSVTQNSGAPGGGISV-S------IR    180
BACOVA_02742    ALGYGIDEVKG-EALTK-AKETNLINSMAGRVPGLVVSQTAGGPSGSTRV-I------LR    183
BACOVA_03426    SVVGAITQTSG-KTLERAGGVTSLGSALTGSLPGVITSASSGMPGAEDPQII------IR    179
BACOVA_03428    DLTHAISTVNT-SELTKKTANNN-------SLVGLESFV-----G------G------YN    79
BACOVA_04393    DLTGSVVSVND-EAIKK-SVVTSVDQVLQGRAAGVQVQANSGMPGGSSSI-R------IR    171
BACOVA_04505    SLTGSVAVVKM-KEVAD-IPTGNVMSGLQGRVAGMNVTTDGKPGGGNTDT-K------LR    171


BT1763          GIGTLN-----------N--NDPLYIIDGVPTKA--------------------GMHE-L    196
BT2264          GVSSLS-----------GT-NQPLYVVDGVPLNNSTVYSTDGL----NSGYDFGNGANAI    133
PG0185 RagA     GTGSLG-----------AS-SAPLYIVDGMQTSL--------------------DVVATM    195
BT0268          GLSSPVFSNK-----DVSS-VQPTVYLNGVPLMLENSYVYDIKQFDINPIGAAANMLAGL    171
BT0272          GVSDISVQSSGDGEFMMGL-TPPLFVVDGIPQEDVTGYDAAG-----LLSGATVSPLAMI    230
BT0362          GIRTMN-----------ASARSPLIIVDNVERD-----------------------LSFL    207
BT3090          GLGTIN-----------N--SNPLVVIDGIPT--------------------DLGLSSL    199
BT3332          GNNSLT-----------QE-NSPLFVIDGFPIED-------------------SSAASTL    191
BT3680          GMSTFG-----------AS-SSALVLIDGIEGN-----------------------INDL    193
BT3702          GGSSLN-----------AS-NDPLIVIDGLAIDNEGI-------------KGMANGLSMV    204
BT3983          GTKSIM-----------QS-SNALYVVDGVPMYSNANKVNGTE----FSSKGNTEPIADI    314
BT4114          GGGSIT-----------GD-NTPLFIVDGFPVES----------------------ISDI    197
BT4121          GISKDG-----------GN-STPLIIIDDVIQLGTNG-------------QPSMEQFNML    220
BT4164          GTSTLN-----------AS-SDPLIVIDGVPYPTEISDDFNFS----TATEEDLGALLNI    230
BT4168          GIRTLT-----------GD-NAPLIVINGVPHMPDKTPSALI-------DGFTRDIFQFY    214
BT4660          GVGTLT-----------GD-SSPLYIVDGFEVDD----------------------IDYL    200
BT4671          GGASLN-----------AS-NDPLIVLDGVPMEVGGS------------ISGGGNFLSLI    215
BACOVA_02096    GTNSFS-----------TN-SQPLYIVDGVPFDTGDTPASDT-----NNSQNKSNPLAFI    228
BACOVA_02652    GINSLN-----------G--NEPLYVIDGVAISGNTD--------------GNSSVLSSI    213
BACOVA_02742    GSTEMT-----------GN-NQPLYVVDGVPLDNTNFGSAGTN-----GGFDLGDGISSI    226
BACOVA_03426    TQSSWN-----------N--SEPLILVDGIERE-----------------------MSSV    203
BACOVA_03428    GSSLWG--------------QGPLVLVDGVPRS-----------------------ASSV    102
BACOVA_04393    GINSLN-----------AS-NEPIFVIDGVIIDGSTG-------------SGSDNALASI    206
BACOVA_04505    GITTIN-----------N--SSPLYVIDGVQTHD--------------------NVASII    198


BT1763          NGNDIESIQVLKDAASASIYGSRAANGVIIITTKQGKKG-QI-KINFDASVSASMYQSKM    254
BT2264          NPDDVANMTILKGAAATALYGSRAANGVVMITTKSGRKE-KGVGIEYNGGVQWSTVLR-L    191
PG0185 RagA     NPNDFESMSVLKDASATSIYGARAANGVVFIQTKKGKMS-ERGRITFNASYGISQILNTK    254
BT0268          DISSIESIEIIKDPLQLAKLGPLAANGAIWITTKDGYYGGE--NVSIGVSAGMAFAPSSV    229
BT0272          PLEDIANIQVLKDAAATSLYGSKGAYGVILIETKRGETA-KP-KVSYSANFVVKTPPRLR    288
BT0362          DAYPIESITILKDAAATAIYGMRGANGAVLVTTKRGETG-KT-KINFTQEVGFQTIAGIP    265
BT3090          NMADVERVDVLKDASATAIYGSRGANGVVMITSKRGAEG-AG-KVTVNANWAIQNATKVP    257
BT3332          NPSDIESLDFLKDASATAIYGARGANGVVIITTKKGKVG-RA-QLSYDGSFGVQHVTRTI    249
BT3680          DPADIESFSILKDASATAVYGTRGANGVVVVTTKRGKAG-KL-HVNFKTNATYSYSPRMP    251
BT3702          NPADIETLTVLKDASATAIYGSRASNGVIIITTKKGKNG-QAPSVSYNGSVSFSKTQKRY    263
BT3983          NPEDIESMSVLTGAAAAALYGSDAANGAIIITTKKGKEG--RVNITVNSNVEFNAPLV-M    371
BT4114          PASDIEDMTVLKDASSTAIYGSRGANGVILVTTKSGKEG-KI-SVNYNAYYSWKKMAKQL    255
BT4121          DPSEVESITVLRDAS-AAIYGSRAANGAILVKTKRGKKG-VP-VISYSGKFAVNDAVSHS    277
BT4164          SPNDIASVEVLKDASATAIWGTQGANGVLVIKTKQGTVG-KT-RFSFSSKWTMKEDPSTI    288
BT4168          HLQDIQNITILKGAE-AAMYGSMGSNGVILIETDGTASNDLETRVSYYGSYGINWNDKRM    273
BT4660          SNSDIESIEVLKDASSSAIYGARAANGVVLITTKSGKTG-RP-TITYNGSASYRKISKKL    258
BT4671          NPNDIESMTVLKDASSTAIYGSRASNGVIIITTKKGSGS-DI-KVSFQTTNSIATKTKTS    273
BACOVA_02096    NPHDIQSIDVLKDASATAIYGSRGANGVVIITTKRGEKG-NE-KVEFSANFTFSKIAKRM    286
BACOVA_02652    NPSDIVSMEILKDASATAIYGSRASNGVVLITTNQGKAS-KT-KVSYEGYYGLQQLPKKL    271
BACOVA_02742    NADDVENMSVLKGPAASALYGSRASHGVILITTKRANKD--KISVEYNGTLTFDTQLAKW    284
BACOVA_03426    DISSVENISVLKDASATAVYGVKGANGVILITTKRGKEG-KA-SVQIKANVTAKVASKLP    261
BACOVA_03428    KASEVESISMLKDAAAVVLYGSRAAKGVILITTKRGKDS--PMHIDVRANVGVNVPKAYP    160
BACOVA_04393    NPSDIVSMDVLKDASATAIYGARAANGVIMITTKRGQKG-EA-QITYDGYIGWQEMPKKL    264
BACOVA_04505    SSNDVESIQVLKDAASAAIYGAQAANGVIIITTKRAKEG-DV-KVSFDMSLTAQTFTGGI    256
```

Supplementary Figure 12 ClustalOmega alignment of SusC orthologs from *B. theta* and *B. ovatus* with known glycan specificities. The RagA protein from *P. gingivalis* (Pg0285)[17] and Bt2264 SusC, a putative peptide importer[15], are shown as well. Only the first ~250 amino acids are shown for clarity, as SusCs are usually >1000 residues in length. The NTE is indicated in blue, with the secondary structure from NMR annotated. Residues corresponding to the Ton

box are shown in red, and those for the plug loop in green (dark green for long plug loops and light green for short plug loops). The plug is coloured purple. X-ray crystal structures have been solved for Bt1763 (no plug loop), Bt2264 (long plug loop; PDB ID 5FQ8) and RagA (no plug loop; PDB ID 6SLN). The alignment shows there are three classes of SusC orthologs, with absent, intermediate and long plug loops.

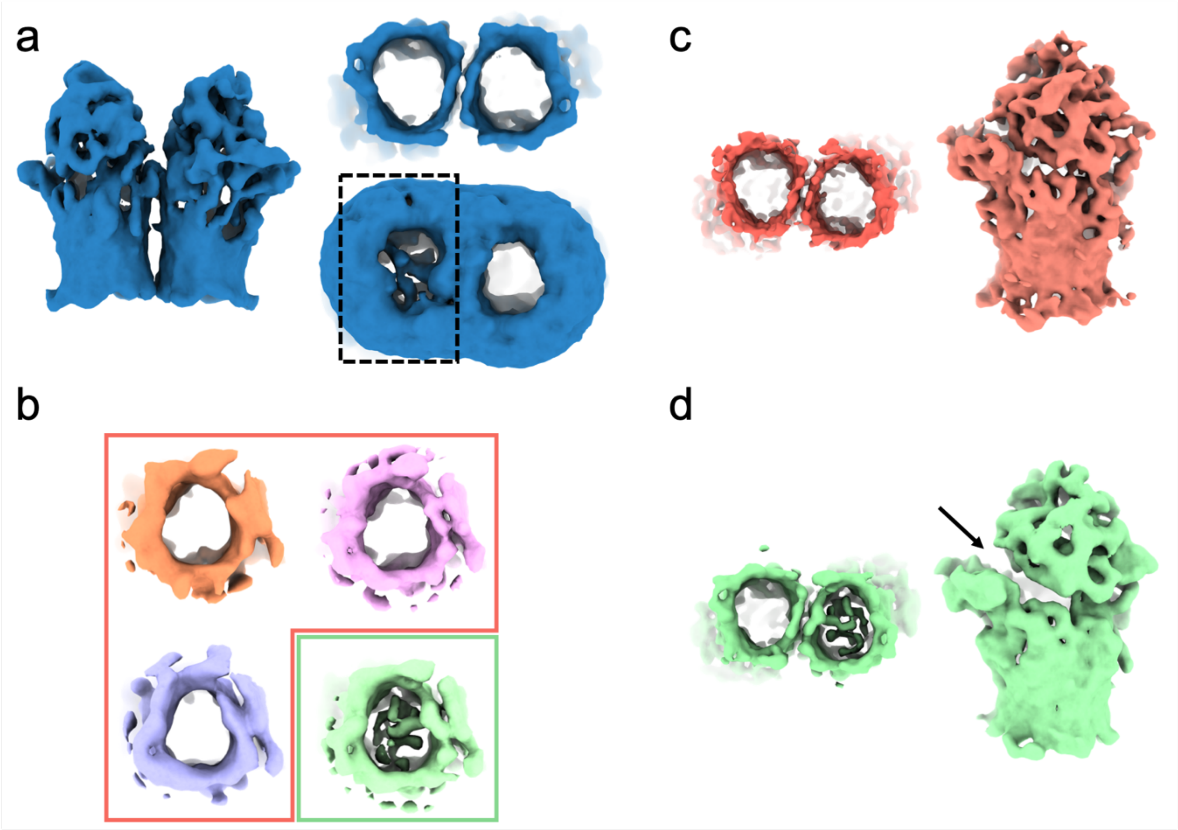

Supplementary Figure 13 Masked classification approach and outputs. **a,** the closed-closed state observed after global 3D classification viewed in the plane of the membrane (left) and from the periplasm (right). Note that at higher contour levels, density corresponding to the plug domain can be observed in just one of the two Bt1763 barrels. Using the volume eraser tool in Chimera, a mask was generated that encompassed the plugged barrel. 'Mask create' in RELION was used to extend this mask by 5 hard and 5 soft pixels. The particle stack contributing to the reconstruction in '**a**' was subjected to 3D classification with the aforementioned mask applied. Alignment was turned off and the T value was set to 20. This classification successfully separated empty and plugged particles (**b**). Empty and plugged particles were then classified again independently in the absence of a mask. The particles contributing to the best-looking classes were refined and post-processed resulting in the reconstructions shown in **c** and **d**: A 'true' closed-closed state lacking density for the plug domain in either barrel (**c**), and a closed-barely open state where clear plug density on only one of the barrels is associated with a partially open position of the Bt1762 lid (**d**).

Supplementary Table 1 X-ray crystallographic data collection and refinement statistics for Bt1762-63

	apo-Bt1763-63	Bt1762-63 + DP15-25	Bt1762-63 + DP6-12
**Data collection**#			
Wavelength (Å)	0.979	0.916	0.978
Space group	$C222_1$	$C222_1$	$C222_1$
Cell dimensions (Å)			
a, *b*, *c* (Å)	116, 233, 171	120, 235,169	120, 238, 171
α, β, γ (°)	90, 90, 90	90, 90, 90	90, 90, 90
Resolution (Å)	96.25-2.62 (2.66-2.62)*	169.2-2.99 (3.04-2.99)	171.0-2.69 (2.74-2.69)
R_{merge}	0.21 (3.08)	0.31 (1.92)	0.22 (1.50)
R_{pim}	0.084 (1.22)	0.13 (0.83)	0.085 (0.61)
$I / \sigma I$	7.0 (0.8)	5.0 (0.7)	6.0 (1.1)
CC1/2	1.00 (0.48)	0.96 (0.63)	0.99 (0.60)
Completeness (%)	99.9 (96.8)	100 (99.3)	100 (100)
Redundancy	7.3 (7.1)	7.2 (6.7)	7.2 (6.9)
Wilson B-factor	52.4	46.7	46.5
**Refinement**			
Resolution (Å)	85.6-2.62	106.6-3.1	97.5-2.69
No. reflections	69,555	43,398	67,654
R_{work} / R_{free} (%)	21.8/27.5	20.6/26.8	20.8/27.5
No. atoms			
Protein	10861	11685	11700
ligands	-	80	175
Water	80	-	243
B-factors			
Protein	66	70	49
ligands	-	77	65
Water	56	-	37
R.m.s. deviations			
Bond lengths (Å)	0.009	0.009	0.008
Bond angles (°)	1.02	1.19	1.01
Clashscore	11.5	13.1	8.2
Ramachandran plot			
Favoured (%)	94.0	90.5	94.1
Outliers (%)	0.7	1.0	0.6
PDB ID	6Z8I	6Z9A	6ZAZ

# One crystal was used for each data collection.
* Values in parentheses are for highest-resolution shell.

Supplementary Table 2 Cryo-EM data collection, refinement and validation statistics for Bt1762-63

	Bt1762-63 (OO) (6ZLT) (EMD-11273)	Bt1762-63 (OC) (6ZM1) (EMD-11277)	Bt1762-63 (CC) (6ZLU) (EMD-11274)
**Data collection and processing**			
Magnification	130,000 x	130,000 x	130,000 x
Voltage (kV)	300	300	300
Electron exposure (e–/Å^2)	63.84	63.84	63.84
Defocus range (μm)	-1.5 to -3.3	-1.5 to -3.3	-1.5 to -3.3
Pixel size (Å)	1.07	1.07	1.07
Symmetry imposed	C2	C1	C2
Initial particle images (no.)		203,450	
Final particle images (no.)	32,190	22,205	17,416
Map resolution (Å)<br>FSC threshold 0.143	3.9	4.7	4.2
Map resolution range (Å)	3.5-5.8	4.3-8.3	3.6-7.5
**Refinement**			
Initial model used (PDB code)	6ZAZ	6ZAZ	6Z8I
Model resolution (Å)<br>FSC threshold (0.143/0.5)	3.4/3.8	4.2/5.8	3.7/7.4
Model resolution range (Å)	∞ - 3.9	∞ - 4.7	∞ - 4.2
Map sharpening *B* factor (Å^2)	-110	-138.78	-92.95
Model composition			
Non-hydrogen atoms	11,425	22,275	10,850
Protein residues	1,485	2,844	1,359
B factors (Å^2)			
Protein (SusC/SusD)	36.20	44.40	52.61
R.m.s. deviations			
Bond lengths (Å)	0.005	0.007	0.008
Bond angles (°)	0.867	0.929	0.988
Validation			
MolProbity score	1.93	2.06	2.08
Clashscore	4.76	13.46	17.04
Poor rotamers (%)	1.90	0.95	0
Ramachandran plot			
Favored (%)	92.30	93.55	94.91
Allowed (%)	7.70	6.24	4.65
Disallowed (%)	0	0.21	0.44

Supplementary Table 3 Heteronuclear experiments and acquisition parameters used for backbone assignments and structure determination of the BT1763 NTE domain.

**Parameter**	**Experiment**					
	2D [^{15}N,^{1}H]-HSQC	2D [^{13}C,^{1}H]-HSQC	3D HNCACB	3D CBCA(CO)NH	3D HHN NOESY	3D HHC NOESY (ali)
^{1}H frequency (MHz)	700	700	700	700	700	700
t_1 data points	300	300	64	120	280	280
t_2 data points	2048	2048	96	100	80	80
t_3 data points	-	-	2048	2048	2048	2048
t_1 max (ms)	52.8	14.2	13.2	5.6	90.9	90.9
t_2 max (ms)	90.9	90.9	4.5	17.6	14	3.78
t_3 max (ms)	-	-	104	91.7	12.4	12.4
SW (F1) (ppm)	40	60	34	60	16	16
SW (F2) (ppm)	16	16	60	40	40	60
SW (F3) (ppm)	-		14	15.94	16	16
F1 carrier (ppm)	117	45	120	45	4.71	4.71
F2 carrier (ppm)	4.71	4.71	45	117	118	45
F3 carrier (ppm)	-	-	4.74	4.71	4.71	4.71
Interscan delay (s)	0.8	0.8	0.8	0.9	0.8	0.8
Number of scans	24	24	8	8	4	4
Mixing time (ms)	-	-	-	-	50	50

Supplementary Table 4 NMR structural statistics of the Bt1763 NTE.

	NTE
**NMR distance and dihedral constraints**	
Distance constraints	
Total	964
Intra-residue ($\|i - j\| = 0$)	281
Inter-residue	683
Sequential ($\|i - j\| = 1$)	294
Medium-range $1<(\|i - j\| < 5)$	70
Long-range ($\|i - j\| > 5$)	319
Hydrogen bonds	0
Total dihedral angle restraints	106
ϕ	53
ψ	53
**Structure statistics**	
Violations (mean and s.d.)	
Distance constraints (Å)	0
Dihedral angle constraints (º)	0
Max. dihedral angle violation (º)	0
Max. distance constraint violation (Å)	0
Ramachandran analysis	
Most favored regions	80.1%
Additionally allowed regions	19.8%
Generously allowed regions	0.1%
Disallowed regions	0.0%
Average r.m.s. deviation (Å)	
Heavy atom	1.03
Backbone	0.52
PDB ID	6YTC

Supplementary Table 5 Affinity of Bt1762-63 for β2,6 FOS determined by ITC.

Ligand[a]	Kd (μM)	ΔG (kJ/mol)	ΔH (kJ/mol)	TΔS (kJ/mol)	N[b]
**DP4 (T346)**	NB[c]	-	-	-	-
**DP5 (T319)**	30.5 (± 24.0)[d]	-25.8	-5.3 (± 1.9)	20.5	1.4 (± 0.2)
**DP6 (T305)**	17.1 (± 3.8)	-27.2	-17.5 (± 1.5)	9.8	1.2 (± 0.04)
**DP8 (T174)**	1.4 (± 0.2)	-33.4	-44.7 (± 1.8)	-11.3	0.8 (± 0.02)
**DP9 (T159)**	1.4 (± 0.2)	-33.5	-29.2 (± 1.7)	4.3	0.9 (± 0.02)
**DP9 (T159) vs W85A[a]**	NB[c]	-	-	-	-
**DP11 (T146)**	1.4 (± 0.2)	-33.5	-30 (± 1.4)	3.5	1.0 (± 0.02)
**~DP12 (T126)**	0.6 (± 0.2)	-35.5	-20.9 (± 1.6)	14.5	1.6 (± 0.04)
**~DP13 (T118)**	0.9 (± 0.2)	-34.6	-41 (± 1.2)	-6.4	1.0 (± 0.02)
**~DP13-14 (T117)**	0.7 (± 0.1)	-35.2	-33.5 (± 1.0)	1.7	1.0 (± 0.01)
**~DP13-14 (T116)**	0.8 (± 0.2)	-34.7	-28.1 (± 1.4)	6.5	1.0 (± 0.02)
**>DP14 (T115)**	1.2 (± 0.1)	-33.8	-30 (± 0.7)	3.3	1.0 (± 0.01)
**>DP16 (T114)**	NB[c]	-	-	-	-
**>DP16 (T113)**	NB[c]	-	-	-	-

[a]DP of major species of FOS as determined by MS is shown. Fraction used (tube, T) is shown in brackets. DP4-6 are from a different FOS purification compared to the larger DP oligosaccharides. All titrations are with wild-type Bt1762-1763 complex (C-term His-tagged Bt1762), except DP9 vs W85A, which is a mutant of Bt1762 in the complex.
[b]N is number of binding sites on the protein.
[c]NB – no binding detected.
[d]The data presented are from single titrations (shown in Figure 6 and Supplementary Figure 9) and errors shown in parentheses are of the fit to the data. In all cases multiple 'scoping' experiments (between 2 to 4 for each ligand) were carried out to determine the optimum concentration of protein and ligand to use for obtaining a saturating binding isotherm (or confirm lack of binding). These scoping runs were all consistent with the final titration data shown here.

Supplementary Table 6 Masses of species observed in the native mass spectra shown in Fig. 7b.

Observed	Assignment Protein + adducts	Number of FOS molecules bound
	4uM protein alone	
177405.37±0.66	$SusC_1D_1$	-
354838.36±4.84	$SusC_2D_2$	-
	4uM protein + 25 µM FOS 159	
177371.32±8.60	SusCD	-
178523.72±60.43	SusCD+1152.4	1
178673.59±19.31	SusCD+1301.7	1
178847.92±14.21	SusCD+1476.6	1
179009.29±18.78	SusCD+1638.0	1
356435.39±83.14	$SusC_2D_2$+1692.75	1
357750.83±3.36	$SusC_2D_2$+3008.19	2
357943.83±9.48	$SusC_2D_2$+3201.19	2
359404.08±18.00	$SusC_2D_2$+4661.44	3
	4uM protein + 50 µM FOS 159	
177380.33±14.22	SusCD	-
178544.08±1.58	SusCD+1163.75	1
178697.68±7.11	SusCD+1317.35	1
178861.85±6.69	SusCD+1481.52	1
179023.06±14.31	SusCD+1642.73	1
356515.81±27.06	$SusC_2D_2$+1677.45	1
357791.44±11.15	$SusC_2D_2$+2953.08	2
357980.53±6.36	$SusC_2D_2$+3219.87	2
359446.38±1.34	$SusC_2D_2$+4685.72	3
360665.22±171.0	$SusC_2D_2$+5826.86	4
	4uM protein + 100 µM FOS 159	
177399.31±9.10	SusCD	-
178543.78±13.78	SusCD+1163.45	1
178710.08±11.09	SusCD+1329.75	1
178878.88±12.17	SusCD+1498.55	1
179046.44±11.71	SusCD+1666.11	1
180366.35±12.78	SusCD+2986.02	2
180203.41±14.05	SusCD+2823.08	2
180672.93±26.94	SusCD+3292.60	2
357823.34±28.59	$SusC_2D_2$+2984.98	2
357987.55±31.87	$SusC_2D_2$+3149.19	2
359488.15±70.86	$SusC_2D_2$+4649.79	3
360839.11±105.09	$SusC_2D_2$+6001.11	4
361151.03±174.06	$SusC_2D_2$+6312.67	4

Supplementary Table 7 Primers used to construct Bt1762-63 SusCD mutants.

Name	Sequence
TB-fwd	AAAGAAGATAACATTCGAGTCGACCTACGGGAGCATG
TBD-rev	CTTTACGCTGTGTAGTATAATCTACCATCTGGGTATC
TBD-fwd	GATACCCAGATGGTAGATTATACTACACAGCGTAAAG
TB-rev	GGTGGCGGCCGCTCTAGAGAGGGTTACGACGGT
TBM-rev	CTTTACGCTGTGTAGTATAAGCAGCAGCAGCAGCAGCATCTACCATCTGGGTATC
TBM-fwd	GATACCCAGATGGTAGATGCTGCTGCTGCTGCTTATACTACACAGCGTAAAG
Hinge-fwd	AAAGAAGATAACATTCGAGTCGACACAATCTTTCCGTCAGCA
H2D-rev	CGAATTTATACCAGTCAGGATATCTGTTGCTTTC
H2D-fwd	ATCCTGACTGGTATAAATTCGGGTGCAATG
Hinge-rev	CACCGCGGTGGCGGCCGCTCTAGAGATCATTCGTTTTATTGA
H1D-rev	TTTGAAACCACCAGGTGCATAGATAGTATAACGGGC
H1D-fwd	TATGCACCTGGTGGTTTCAAACGCAACCAGATCGGT
Trp1-fwd	AAAGAAGATAACATTCGAGTCGACGAAGAGTGTAGTCGGACATAC
Trp2-rev	ATTGATATTCGCGTCGGTGGTATTGATACC
Trp3-fwd	ACCACCGACGCGAATATCAATGATATATGG
Trp4-rev	CACCGCGGTGGCGGCCGCTCTAGACTGAATCAGTGCTTCGGCAC
NTE1-fwd	AAAGAAGATAACATTCGAGTCGACCTACGGGAGCATG
NTE2-rev	AACTTCATCTACCATCTGAATGTTTTGCCCCCACAG
NTE3-fwd	CTGTGGGGGCAAAACATTCAGATGGTAGATGAAGTT
NTE4-rev	GGTGGCGGCCGCTCTAGAGAGGGTTACGACGGT
