# Supplementary material for: Insights into SusCD-mediated glycan import by a prominent gut symbiont
Source: Nat Commun. 2021 Jan 4;12:44. doi: 10.1038/s41467-020-20285-y (PMC7782687; doi:10.1038/s41467-020-20285-y)
Supplement: Supplementary file 3 — Reporting Summary [file 41467_2020_20285_MOESM3_ESM.pdf]

nature research

Corresponding author(s): David Bolam, Bert van den Berg

Last updated by author(s): November 12, 2020

Reporting Summary

Nature Research wishes to improve the reproducibility of the work that we publish. This form provides structure for consistency and transparency in reporting. For further information on Nature Research policies, see Authors & Referees and the Editorial Policy Checklist.

Statistics

For all statistical analyses, confirm that the following items are present in the figure legend, table legend, main text, or Methods section.

n/a	Confirmed	
☐	☒	The exact sample size (*n*) for each experimental group/condition, given as a discrete number and unit of measurement
☐	☒	A statement on whether measurements were taken from distinct samples or whether the same sample was measured repeatedly
☒	☐	The statistical test(s) used AND whether they are one- or two-sided<br>*Only common tests should be described solely by name; describe more complex techniques in the Methods section.*
☒	☐	A description of all covariates tested
☒	☐	A description of any assumptions or corrections, such as tests of normality and adjustment for multiple comparisons
☐	☒	A full description of the statistical parameters including central tendency (e.g. means) or other basic estimates (e.g. regression coefficient) AND variation (e.g. standard deviation) or associated estimates of uncertainty (e.g. confidence intervals)
☒	☐	For null hypothesis testing, the test statistic (e.g. *F*, *t*, *r*) with confidence intervals, effect sizes, degrees of freedom and *P* value noted<br>*Give P values as exact values whenever suitable.*
☒	☐	For Bayesian analysis, information on the choice of priors and Markov chain Monte Carlo settings
☒	☐	For hierarchical and complex designs, identification of the appropriate level for tests and full reporting of outcomes
☒	☐	Estimates of effect sizes (e.g. Cohen's *d*, Pearson's *r*), indicating how they were calculated

Our web collection on statistics for biologists contains articles on many of the points above.

Software and code

Policy information about availability of computer code

Data collection

For CryoEM data collection:
ThermoFisher:
EPU 1.8
TEM software (2.9.1)
TEM Imaging Analysis (TIA)(4.17)
FluCam (6.9.1)
AutoCTF (0.6.9)

GATAN:
GMS 3/Digital Micrograph (DM)(3.22)

For MS data acquisition:
Tune software, ThermoFischer

ITC: Microcal PEAQ-ITC control software v1.21 (Malvern Panalytical)

For NMR data collection:
Bruker Avance 700
Topspin 3.6 software

Data analysis

For CryoEM data analysis and structure refinement:
RELION v2.1
RELION v3.0
Motioncorr v2

gCTF v1.06

X-ray data processing and refinement:
Xia2 0.6.467
DIALS 3.1
Pointless 1.12.2
Aimless 0.7.4
Phenix version 1.18-3855 (includes Phaser)
Coot 0.9

Making figures: GraphPad Prism version 7.

Mass Spec data visualization and processing:
Xcalibur v2.2, Thermo Fischer
Origin v9.2, OriginLab

ITC: MicroCal PEAQ ITC Analysis Software v1.30 (Malvern Panalytical)

For NMR data analysis and structure calculation:
CARA 1.9.1.7
CYANA 3.98.5

For manuscripts utilizing custom algorithms or software that are central to the research but not yet described in published literature, software must be made available to editors/reviewers. We strongly encourage code deposition in a community repository (e.g. GitHub). See the Nature Research guidelines for submitting code & software for further information.

Data

Policy information about availability of data

All manuscripts must include a data availability statement. This statement should provide the following information, where applicable:

- Accession codes, unique identifiers, or web links for publicly available datasets
- A list of figures that have associated raw data
- A description of any restrictions on data availability

The data supporting the findings of this study are available from the corresponding authors upon reasonable request. Coordinates and structure factors that support the findings of this study have been deposited in the Protein Data Bank with accession codes 6ZAZ [http://doi.org/10.2210/pdb6ZAZ/pdb] (Bt1762-63 with shorter FOS), 6Z8I [http://doi.org/10.2210/pdb6Z8I/pdb] (Bt1762-63 apo) and 6Z9A [http://doi.org/10.2210/pdb6Z9A/pdb] (Bt1762-63 with longer FOS). EM structure coordinates have been deposited in the Electron Microscopy Data Bank with accession codes 6ZLT [https://doi.org/10.2210/pdb6ZLT/pdb] (OO; EMD-11273 [https://www.ebi.ac.uk/pdbe/entry/emdb/EMD-11273]), 6ZM1 [https://doi.org/10.2210/pdb6ZM1/pdb] (OC; EMD-11277 [https://www.ebi.ac.uk/pdbe/entry/emdb/EMD-11277]) and 6ZLU [https://doi.org/10.2210/pdb6ZLU/pdb] (CC; EMD-11274 [https://www.ebi.ac.uk/pdbe/entry/emdb/EMD-11274]). The NMR data have been deposited and publicly released in the Biological Magnetic Resonance Bank (BMRB) with number 34514 [https://bmrb.io/data_library/summary/index.php?bmrbId=34514]. The NTE structure has been deposited in the Protein Data Bank with accession code 6YTC [http://doi.org/10.2210/pdb6YTC/pdb]. Source data are provided with this paper.

Field-specific reporting

Please select the one below that is the best fit for your research. If you are not sure, read the appropriate sections before making your selection.

☒ Life sciences ☐ Behavioural & social sciences ☐ Ecological, evolutionary & environmental sciences

For a reference copy of the document with all sections, see nature.com/documents/nr-reporting-summary-flat.pdf

Life sciences study design

All studies must disclose on these points even when the disclosure is negative.

Sample size	No sample size calculation was performed. For bacterial growths and ITC experiments the sample size used was based on the level of variability within the replicates performed and previous experience with these techniques.
Data exclusions	No data exclusion was needed.
Replication	The replicas of data gave similar results. Bacterial growths are averages of triplicate wells in the plater reader and are representative of 3 independent experiments. All ITC runs were repeated between 2 and 4 times depending on the ligand tested and gave consistent results each time.
Randomization	This is not relevant to our study, because no grouping was needed.
Blinding	Investigators were not blinded to group allocation, as no grouping was needed for this study.

Reporting for specific materials, systems and methods

We require information from authors about some types of materials, experimental systems and methods used in many studies. Here, indicate whether each material, system or method listed is relevant to your study. If you are not sure if a list item applies to your research, read the appropriate section before selecting a response.

Materials & experimental systems

n/a	Involved in the study
☒	☐ Antibodies
☒	☐ Eukaryotic cell lines
☒	☐ Palaeontology
☒	☐ Animals and other organisms
☒	☐ Human research participants
☒	☐ Clinical data

Methods

n/a	Involved in the study
☒	☐ ChIP-seq
☒	☐ Flow cytometry
☒	☐ MRI-based neuroimaging
